# Epidemiology of hip fractures in Bulgaria: development of a country-specific FRAX model

**DOI:** 10.1007/s11657-020-0710-2

**Published:** 2020-02-27

**Authors:** E. Kirilova, H. Johansson, N. Kirilov, S. Vladeva, T. Petranova, Z. Kolarov, E. Liu, M. Lorentzon, L. Vandenput, N. C. Harvey, E. McCloskey, John A. Kanis

**Affiliations:** 1 Medical University of Sofia, Sofia, Bulgaria; 2 Mary MacKillop Institute for Health Research, Australian Catholic University, Melbourne, Australia; 3 Medical Faculty, Trakia University, Stara Zagora, Bulgaria; 4 Geriatric Medicine, Institute of Medicine, University of Gothenburg, Gothenburg, Sweden; 5 Centre for Bone and Arthritis Research, Institute of Medicine, University of Gothenburg, Gothenburg, Sweden; 6 MRC Lifecourse Epidemiology Unit, University of Southampton, Southampton, UK; 7 Centre for Metabolic Bone Diseases, University of Sheffield, Sheffield, UK

**Keywords:** Bulgaria, FRAX, Fracture probability, Epidemiology, Hip fracture

## Abstract

**Abstract:**

***Summary*:**

A retrospective population-based survey was undertaken in a region of Bulgaria to determine the incidence of hip fracture. The estimated number of hip fractures nationwide for 2015 was 9322 and is predicted to increase to 11,398 in 2050. The hip fracture rates were used to create a FRAX model.

**Objective:**

To describe the epidemiology of hip fractures in Bulgaria, which was then used to develop the country-specific fracture prediction FRAX® tool.

**Methods:**

We carried out a retrospective population-based survey in Stara Zagora, Bulgaria, representing approximately 4.6% of the country’s population. We identified hip fractures occurring in 2015, 2016 and 2017 from hospital registers and primary care sources held by the regional health insurance agency. Age- and sex-specific incidence of hip fracture and national mortality rates were incorporated into a FRAX model for Bulgaria. Fracture probabilities were compared with those from neighbouring countries having FRAX models.

**Results:**

The incidence of hip fracture applied nationally suggested that the estimated number of hip fractures nationwide in persons over the age of 50 years for 2015 was 9322 and is predicted to increase to 11,398 in 2050. FRAX-based probabilities were higher in Bulgaria than those in Serbia or Romania, lower than those in Turkey and similar to those in Greece.

**Conclusion:**

The FRAX model should enhance accuracy of determining fracture probability among the Bulgarian population and help guide decisions about treatment.

## Introduction

Osteoporosis and osteoporotic fractures are becoming more common with advancing age. In Europe, the annual cost of fractures associated with osteoporosis exceeded € 37 billion in 2010 [1], and disability due to osteoporosis was greater than that caused by any single cancer, with the exception of lung cancer and was comparable or greater than that lost to a variety of chronic non-communicable diseases, such as rheumatoid arthritis, asthma and high blood pressure–related heart disease [2]. Fortunately, a wide variety of treatments is available that favourably affect bone mass and thereby decrease the risk of fractures associated with osteoporosis [3]. The use of such interventions by healthcare practitioners is assisted by instruments that assess patients’ fracture risk to optimise clinical decisions about prevention and treatment. The most widely used web-based tool FRAX® (https://www.sheffield.ac.uk/FRAX/) meets these requirements and computes the 10-year probability of fragility fractures based on several common clinical risk factors and optionally, a DXA scan result [4, 5]. FRAX models are available for 66 countries covering more than 80% of the world population at risk [6] and have been incorporated into more than 100 guidelines worldwide [7].

The availability of FRAX has stimulated studies for the generation of new FRAX models. Specific examples include Brazil, Mexico, Turkey [8–10] and several countries in Eastern Europe [11–14]. The present report describes the epidemiology of fractures at the hip in Bulgaria and the generation of a country-specific FRAX model.

## Methods

Bulgaria is a country in Southeastern Europe with a total area of 110,994 km^2^. It borders the Black Sea to the east, Greece and Turkey to the south, Serbia and Macedonia to the west and Romania to the north [15]. According to the national statistical institute, the Bulgarian population comprises four ethnic groups as follows: Bulgarian (84.8%), Turkish (8.8%), Roma (4.9%) and others (1.5%) [16].

Stara Zagora, in Southern Bulgaria, is the sixth-largest city in Bulgaria. Based on regional health inspection’s data, the region’s population aged 40 years or above in 2017 amounted to 183,294 people (84,557 men and 98,737 women) and represented 4.6% of the whole Bulgarian population aged 40 years and above (183,294/3,982,770). The ethnic admixture of Stara Zagora is similar to the country as a whole.

We analysed the cases of hip fractures (ICD codes: S72.0, S72.1 and S72.2) in the Stara Zagora region, Bulgaria, for the years 2015, 2016 and 2017. Data were provided by the Regional Health Insurance Fund (RHIF) Stara Zagora using an electronic database of hospitals, general practitioners and specialists in this region. The insurance company covers 81.2% of the total population but coverage varies according to age and sex. The relevant data on coverage was available and the age- and sex-specific insured population comprised the denominator for the calculation of incidence. To minimise double counting, further admissions for the same hip fracture site within 3 months were excluded. Permanent residence in the region was not a criterion for inclusion, so a small number of patients living temporarily in the catchment area were also included in the database.

Incidence of hip fractures was calculated from the age of 40 years in 5-year age intervals in men and in women. The age of 40 was chosen since FRAX permits the calculation of fracture probability from this age.

The age- and sex-specific incidence for 2015–2017 was applied to the Bulgarian population for 2015 to estimate the number of hip fractures nationwide. Additionally, future projections were estimated up to 2050, assuming that the age- and sex-specific incidence remained stable. Population demography was taken from the United Nations using the medium variant for fertility [17].

The data on hip fracture were used to construct a FRAX model for Bulgaria. For other major osteoporotic fractures (clinical spine, forearm and humeral fractures), it was assumed that the age- and sex-specific ratios of these fractures to hip fracture risk in Bulgaria were comparable with those found in Sweden [4]. This assumption has been used for many of the FRAX models with incomplete epidemiological information on non-hip fractures. Available information suggests that the age- and sex-stratified pattern of fracture is very similar in the Western world and Australia [14, 18–20].

The development and validation of FRAX have been extensively described [4, 5]. The risk factors used were based on a systematic set of meta-analyses of population-based cohorts worldwide and validated in independent cohorts with over 1 million patient-years of follow-up. The construct of the FRAX model for Bulgaria retained the beta coefficients of the risk factors in the original FRAX model with the incidence rates of hip fracture and mortality rates for Bulgaria. National mortality rates used data from the United Nations in 2014 [21]. Ten-year fracture probabilities were compared with those of neighbouring countries with FRAX models (Greece, Serbia, Turkey and Romania).

In order to compare Bulgarian hip fracture probabilities with those of other regions of the world, the remaining lifetime probability of hip fracture from the age of 50 years was calculated for men and women as described by Kanis et al. [22]. In the present analysis, values for Bulgaria were compared with those of China (with and without inclusion of Hong Kong), Canada, Denmark, Finland, France, Greece, Hungary, Portugal, Spain, Sweden, UK and the USA [22], with more recent additions from Mexico [23], Romania [24], Poland [25], Moldova [14], Russia [12], Turkey [10], Ukraine [26] and Serbia [27].

## Results

A total of 367 hip fractures were identified in men and 1017 in women (female/male ratio 2.8). Below the age of 65 years, hip fractures were more prevalent in men than in women (female/male ratio 0.7), but thereafter were more frequent in women (female/male ratio 3.2). The incidence of hip fracture increased with age in men and women, though more markedly in women (Table 1).

**Table 1:** Population of the catchment area, number of hip fractures and annual incidence of hip fractures (rate/100,000) in men and women in Stara Zagora, Bulgaria, by age for years 2015, 2016 and 2017 combined

Age (years)	Population^a^	Fractures^b^	Incidence/100,000	95% CI
Men				
40–44	28,257	9	32	15–60
45–49	27,267	14	51	28–86
50–54	26,372	18	68	40–108
55–59	27,646	26	94	61–138
60–64	27,889	35	125	87–175
65–69	27,903	37	133	93–183
70–74	20,886	44	211	153–283
75–79	15,632	54	345	259–451
80–84	11,131	54	485	364–633
85–89	4832	53	1097	822–1435
90+	1292	23	1780	1128–2672
40+	219,106	367	167	151–186
Women				
40–44	28,069	3	11	2–31
45–49	26,998	7	26	10–53
50–54	27,768	12	43	22–76
55–59	30,383	24	79	51–118
0–64	34,907	36	103	72–143
65–69	38,011	60	158	120–203
70–74	30,842	95	308	249–377
75–79	24,652	189	767	661–884
80–84	19,278	261	1354	1195–1529
85–89	9247	247	2671	2348–3026
90+	2862	83	2900	2310–3595
40+	273,018	1017	373	350–396

^a^Population corrected for insurance cover

^b^Fractures per year over 3 years

### Hip fracture projections

Assuming that the fracture rates in Stara Zagora were representative for the whole country and based on the UN estimates of the Bulgarian population for 2015, we estimated that the annual number of hip fractures in men and women aged 50 years and older in Bulgaria in 2015 was 9322, comprising 2521 in men and 6801 fractures in women. The number of hip fractures is expected to increase progressively by calendar year with an increase of 22% by 2050 (Table 2). The increase in hip fracture numbers was higher in women (24% in women and 18% in men) due to the high age dependency of hip fracture incidence.

**Table 2:** Estimated total number of hip fractures (ICD-10 codes S72.0, S72.1, S72.2) in men and in women aged 50 years and older in 2015 projected up to 2050 in Bulgaria

	2015	2020	2030	2040	2050
Men	2521	2567	2739	2903	2973
Women	6801	7136	7923	8397	8425
Total	9322	9703	10,662	11,300	11,398
Increase (%)	-	4	14	21	22

### Fracture probability

The 10-year probability of major osteoporotic fracture and hip fracture in Bulgaria and neighbouring countries is shown in Table 3 in women with a prior fracture by age. Ten-year probabilities were consistently higher than those in the neighbouring countries of Serbia and Romania, lower than those in Turkey and similar to those in Greece.

**Table 3:** Ten-year probability of a major osteoporotic fracture (MOF) and hip fracture in women with a prior fracture by age from Bulgaria, Greece, Turkey, Serbia and Romania. Body mass index set to 25 kg/m^2^

	Bulgaria	Greece	Turkey	Serbia	Romania
MOF					
50	7.7	5.2	8.2	4.5	5.2
55	8.5	7.0	8.2	6.1	6.5
60	9.5	9.8	8.6	8.4	7.9
65	11	13	10	11	9.5
70	15	17	13	13	11
75	19	22	18	14	13
80	22	25	24	15	13
85	23	25	33	15	13
90	20	20	37	14	11
Hip					
50	1.2	0.8	1.3	0.7	0.8
55	1.6	1.3	1.5	1.2	1.2
60	2.1	2.2	1.8	1.9	1.8
65	2.9	3.4	2.3	2.9	2.6
70	4.5	5.4	4.1	4.2	3.5
75	7.0	8.3	7.5	5.3	4.6
80	9.6	11	11	5.8	5.4
85	11	12	16	6.7	5.8
90	9.4	9.3	17	7.3	4.5

For the remaining lifetime probability of a hip fracture in Bulgarian women from the age of 50 years was 11.2% which lay midway between Romania and Serbia (7.1 and 7.7%, respectively) and those of Greece and Turkey (15.4 and 15.9%, respectively) (Table 4).

**Table 4:** Lifetime probability of hip fracture in the Bulgarian population from the age of 50 years compared with selected countries

Country	Lifetime risk at 50 years (%)^a^	
	Women	Men
Sweden	25.6	11.0
Denmark	23.0	11.3
France	19.3	5.9
China (Hong Kong)	17.7	7.6
USA (Caucasian)	16.1	7.5
Turkey	15.9	3.6
Canada	15.5	5.8
Greece	15.4	6.8
UK	14.4	5.0
Portugal	13.7	4.8
Finland	12.9	6.0
Spain	12.6	4.2
Bulgaria^b^	11.2	4.4
Hungary	10.8	4.2
Mexico	10.6	5.0
Poland	10.1	4.2
Moldova	9.3	5.7
Russia	7.7	3.8
Serbia	7.7	3.7
Romania	7.1	3.8
China	5.9	3.3
Ukraine	5.6	2.9

^a^Probabilities derived from fracture as given in FRAX v4.1 and updated death risks

^b^Present study

## Discussion

The present study characterised the regional incidence of hip fracture in a well-defined catchment area. The hip fracture incidence increased with age in both sexes, but below the age of 65 years was higher in men than in women. Thereafter, incidence was higher in women. Similar results have been reported in many studies including other countries in Eastern Europe, namely Russia [12], Armenia [11], Moldova [14] and Belarus, [13]. From these results, Bulgaria belongs to the moderate risk countries for osteoporotic hip fracture for men and women [28].

Based on the regional incidence, the number of hip fractures in 2015 was estimated at 9322 for the whole country and is expected to increase by 22% to 11,398 in 2050. These estimates are relatively robust, in that all individuals who will be aged 60 years or more in 2050 are currently adults. However, these estimates may be conservative since they assume that the age- and sex-specific risk of hip fracture remains unchanged over this period. Decreases in age-specific rates have occurred in those countries with the higher hip fracture risks [29], whereas increases in incidence with time are commonly found in those countries with the lower risks. It is estimated that modest increases in secular trends (e.g. 1% per year) as seen for example in Mexico [9] together with demographic changes would double the number of hip fractures over 20 years [30]. Such projections are important for healthcare planning.

The incidence of hip fracture and of death was used to create a country-specific FRAX model for Bulgaria. Ten-year probabilities of fracture were consistently higher than those in the neighbouring countries of Serbia and Romania, lower than those in Turkey and similar to those in Greece. These differences in fracture probability cannot be accounted for by differences in mortality but rather reflect differences in the risk of hip fracture. Reasons for the heterogeneity in hip fracture risk are speculative [9]. The factor which best predicts the heterogeneity in hip fracture risk is socioeconomic prosperity that in turn may be related to low levels of physical activity [31]. The fact that there are differences in adjacent countries emphasizes the importance of the use of country-specific FRAX models rather than surrogate models [32].

A minority of countries that have a FRAX model also have robust information on the risk of other major osteoporotic fractures. In the absence of such information, FRAX models are based on the assumption that the age- and sex-specific pattern of these fractures is similar to that observed in Malmo, Sweden [33]. The acquisition of data on the incidence of forearm and humerus fractures in a manner identical to that for hip fracture permitted the adequacy of this assumption to be tested, at least for forearm and humeral fractures. Our findings suggest that the incidence of forearm and humerus fractures can be reasonably predicted from the incidence of hip fracture. Very similar findings have been reported from Canada [20], Iceland [19], USA [34], UK [35], Australia [36] and several additional countries of the Western world and Australia, despite differences in incidence [28]. This commonality of pattern is supported by register studies, which indicate that in those regions where hip fracture rates are high, so too is the risk of forearm fracture and spine fractures (requiring hospital admission) [37, 38].

In most countries, a case finding approach is used for the management of osteoporosis, where certain clinical risk factors (CRFs) for fracture suggest the possible diagnosis of osteoporosis and trigger a more detailed assessment of the need for intervention. Many assessment guidelines recommend that women with a prior fracture are eligible for treatment. By the same token, individuals with a fracture probability that is equivalent to or greater than that of women with a prior fracture should also be eligible. Age-specific intervention thresholds have been widely used in Europe and South America [7]. If the same strategy were used in Bulgaria, the intervention would be recommended in individuals with a 10-year probability of a major osteoporotic fracture that ranged from 7.7 to 23%, depending on age (see Table 3).

There are a number of limitations to this study. The accuracy of the register is not known. With regard to fracture incidence, we examined somewhat less than 5% of the Bulgarian population. Therefore, the extrapolation of these regional estimations to the entire country is an assumption that we were unable to test. This would require a national survey and its validity tested in prospective study of a population-based cohort. Moreover, a minority of the population was not included in the regional health insurance fund and an imbalance in health status may have skewed our estimates. In addition to large variations in fracture rates around the world, fracture rates may vary within countries. In addition to ethnic-specific differences [39], up to twofold differences in hip fracture incidence have been reported using common methodology with the higher rates in urban communities including Croatia [40], Switzerland [41], Norway [42], Argentina [43] and Turkey [44].

Despite the well-defined catchment population, it is possible that not all hip fractures were captured. Indeed, many patients in Eastern Europe are not hospitalized because facilities for surgical management are limited, so that hospital admission is not always feasible. In Belarus, for example, 29% cases of hip fracture did not come to hospital attention [13]. High rates of non-admittance have been reported in Armenia (44%) [11] Pervouralsk in Russia (27%) [12], Georgia (75%) and Kyrgyzstan (50%) [45].

It is relevant, however, that accuracy errors have little impact on the rank order with which the FRAX tool categorises risk in a given population [11, 46], but they do change the absolute number generated and thus have implications where treatment guidelines are based on cost-effectiveness or the economic burden of disease. In order to address these limitations, population representatives of the general population at risk would need to be studied prospectively, preferably over a 10-year time horizon.

In summary, a FRAX model has been created for Bulgaria based on a regional population-based estimate of the incidence of low energy hip fractures. The model should enhance accuracy of determining fracture probability among the Bulgarian population and help to guide decisions about treatment.

## References

[1] Hernlund E, Svedbom A, Ivergård M, Compston J, Cooper C, Stenmark J, McCloskey EV, Jönsson B, Kanis JA (2013). Osteoporosis in the European Union: medical management, epidemiology and economic burden. A report prepared in collaboration with the International Osteoporosis Foundation (IOF) and the European Federation of Pharmaceutical Industry Associations (EFPIA). Arch Osteoporos.

[2] Johnell O, Kanis JA (2006). An estimate of the worldwide prevalence and disability associated with osteoporotic fractures. Osteoporos Int.

[3] Kanis JA, Cooper C, Rizzoli R, Reginster J-Y, Scientific Advisory Board of the European Society for Clinical and Economic Aspects of Osteoporosis (ESCEO) and the Committees of Scientific Advisors and National Societies of the International Osteoporosis Foundation (IOF) (2019). European guidance for the diagnosis and management of osteoporosis in postmenopausal women. Osteoporos Int.

[4] Kanis JA On behalf of the World Health Organization Scientific Group (2008a) Assessment of osteoporosis at the primary healthcare level. Technical Report. WHO Collaborating Centre, University of Sheffield, UK. Available at http://www.shef.ac.uk/FRAX/pdfs/WHO_Technical_Report.pdf. Accessed 26 Feb 2019

[5] Kanis JA, Johnell O, Oden A, Johansson H, McCloskey E (2008). FRAX™ and the assessment of fracture probability in men and women from the UK. Osteoporos Int.

[6] Odén A, McCloskey EV, Kanis JA, Harvey N, Johansson H (2015). Burden of high fracture probability. Osteoporos Int.

[7] Kanis JA, Harvey NC, Cyrus Cooper C, Johansson H, Odén A, McCloskey EV, The Advisory Board of the National Osteoporosis Guideline Group (2016). A systematic review of intervention thresholds based on FRAX. A report prepared for the National Osteoporosis Guideline Group and the International Osteoporosis Foundation. Arch Osteoporos.

[8] Zerbini CAF, Szejnfeld VL, Abergaria BH, McCloskey EV, Johansson H, Kanis JA (2015). Incidence of hip fracture in Brazil and the development of a FRAX model. Arch Osteoporos.

[9] Johansson H, Clark P, Carlos F, Oden A, McCloskey EV, Kanis JA (2011). Increasing age- and sex-specific rates of hip fracture in Mexico: a survey of the Mexican institute of social security. Osteoporos Int.

[10] Tuzun S, Eskiyurt N, Akarırmak U, Sarıdoğan M, Johansson H, Kanis JA, Turkish Osteoporosis Society (2012). The impact of FRAX-based intervention thresholds in Turkey: the FRAX-TURK study. Arch Osteoporos.

[11] Lesnyak O, Sahakyan S, Zakroyeva A, Bilezikian JP, Hutchings N, Galstyan R, Lebedev A, Johansson H, Harvey NC, McCloskey E, Kanis JA (2017). Epidemiology of fractures in Armenia: development of a country-specific FRAX model and comparison to its surrogate. Arch Osteoporos.

[12] Lesnyak O, Ershova O, Belova K, Gladkova E, Sinitsina O, Ganert O, Romanova M, Khodirev V, Johansson H, McCloskey E, Kanis JA (2012). Epidemiology of fracture in the Russian Federation and the development of a FRAX model. Arch Osteoporos.

[13] Ramanau H, Chernyanin I, Rudenka E, Lesnyak O, Zakroyeva A, Bilezikian JP, Johansson H, Harvey NC, McCloskey EV, Kanis JA (2018). Epidemiology of hip fracture in Belarus: development of a country-specific FRAX model and its comparison to neighboring country models. Arch Osteoporos.

[14] Zakroyeva A, Lesnyak O, Cazac V, Groppa L, Russu E, Chislari L, Rotaru L, Johansson H, Harvey NC, McCloskey E, Kanis JA (2019) Epidemiology of osteoporotic fracture in Moldova and development of a country specific FRAX model. Submitted to Archives June 2019 Minor revisions and resubmitted (Sept 2019) 10.1007/s11657-019-0669-z PMC6987067 31993755

[15] Bulgaria doing business for everyone guide-Practical Information and Contacts, 2013 Edition Updated Reprint International Business Publication, USA ISBN 1-4387-7175-4 89

[16] National statistical institute republic Bulgaria census2011/PDOCS2/Census2011. http://www.nsi.bg/census2011/PDOCS2/Census2011final_en.pdf

[17] United Nations (2017) DESA/population division. World Population Prospects 2017 https://population.un.org/wpp/Download/Standard/Population/. Accessed 15 Feb 2019

[18] Kanis JA, Oden A, Johnell O, Jonsson B, de Laet C, Dawson A (2001). The burden of osteoporotic fractures: a method for setting intervention thresholds. Osteoporos Int.

[19] Siggeirsdottir K, Aspelund T, Johansson H, Gudmundsson EF, Mogensen B, Jonsson BY, Gudnason V, McCloskey E, Oden A, Sigurdsson G, Kanis JA (2014). The incidence of a first major osteoporotic fracture in Iceland and implications for FRAX. Osteoporos Int.

[20] Lam A, LeslieWD LLM, Yogendran M, Morin SN, Majumdar SR (2014). Major osteoporotic to hip fracture ratios in Canadian men and women with Swedish comparisons: a population-based analysis. J Bone Miner Res.

[21] United Nations (2010) Population Division of the Department of Economic and Social Affairs of the United Nations Secretariat, World Population Prospects: http://esa.un.org/unpd/wpp/unpp/panel_indicators.htm Accessed Nov 2011

[22] Kanis JA, Johnell O, De Laet C, Jonsson B, Oden A, Ogelsby AK (2002). International variations in hip fracture probabilities: implications for risk assessment. J Bone Miner Res.

[23] Clark P, Lavielle P, Franco-Morina F, Ramirez E, Salmeron J, Kanis JA, Cummings SR (2005). Incidence rates and life-time risk of hip fractures in Mexicans over 50 years of age: a population-based study. Osteoporos Int.

[24] Grigorie D, Sucaliuc A, Johansson H, Kanis JA, McCloskey E (2013). Incidence of hip fracture in Romania and the development of a Romanian FRAX model. Calcif Tiss Int.

[25] Czerwinski E, Kanis JA, Trybulec B, Johansson H, Borowy P, Osieleniec J (2009). The incidence and risk of hip fracture in Poland. Osteoporos Int.

[26] Povoroznyuk VV, Grygorieva NV, Kanis JA, McCloskey EV, Johansson H, Harvey NC, Korzh MO, Strafun SS, Vaida VM, Klymovytsky FV, Vlasenko RO, Forosenko VS (2017). Epidemiology of hip fracture and the development of FRAX in Ukraine. Arch Osteoporos.

[27] Johansson H, Matijevic R, Harhaji V, McCloskey EV, Harvey NC, Lorentzon M, Liu E, Kanis JA (2019). Ten-year fracture probability in Serbia according to age and other FRAX models in the region. Osteoporos Int.

[28] Kanis JA, Oden A, McCloskey EV, Johansson HD, Wahl A, Cooper C (2012). A systematic review of hip fracture incidence and probability of fracture worldwide. Osteoporos Int.

[29] Cooper C, Cole ZA, Holroyd CR, Earl SC, Harvey NC, Dennison EM, Melton LJ, Cummings SR, Kanis JA, IOF CSA Working Group on Fracture Epidemiology (2011). Secular trends in the incidence of hip and other osteoporotic fractures. Osteoporos Int.

[30] Gullberg B, Johnell O, Kanis JA (1997). World-wide projections for hip fracture. Osteoporos Int.

[31] Johnell O, Borgstrom F, Jonsson B, Kanis J (2007). Latitude, socioeconomic prosperity, mobile phones and hip fracture risk. Osteoporos Int.

[32] Cauley JA, El-Hajj Fuleihan G, Arabi A, Fujiwara S, Ragi-Eis S, Calderon A, Chionh SB, Chen Z, Curtis JR, Danielson ME, Hanley DA, Kroger H, Kung AW, Lesnyak O, Nieves J, Pluskiewicz W, El Rassi R, Silverman S, Schott AM, Rizzoli R, Luckey M, FRAX® Position Conference Members (2011). Official positions for FRAX clinical regarding international differences from Joint Official Positions Development Conference of the International Society for Clinical Densitometry and International Osteoporosis Foundation on FRAX. J Clin Densitom.

[33] Kanis JA, Hans D, Cooper C, Baim S, Bilezikian JP, Binkley N, Compston J, Dawson-Hughes B, El-Hajj Fuleihan G, Johansson H, Leslie WD, Lewiecki EM, Luckey MM, Oden A, Papapoulos SE, Poiana C, Wahl DA, McCloskey E, Task Force of the FRAX Initiative (2011). Interpretation and use of FRAX in clinical practice. Osteoporos Int.

[34] Melton LJ, Crowson CS, O’Fallon WM (1999). Fracture incidence in Olmsted County, Minnesota: comparison of urban and with rural rates and changes in urban rates over time. Osteoporos Int.

[35] Singer BR, McLauchlan CJ, Robinson CM, Christie J (1998). Epidemiology of fracture in 15.000 adults. The influence of age and gender. J Bone Joint Surg.

[36] Sanders KM, Seeman E, Ugoni AM, Pasco JA, Martin TJ, Skoric B, Nicholson GC, Kotowicz MA (1999). Age- and gender-specific rate of fractures in Australia: a population-based study. Osteoporos Int.

[37] Johnell O, Gullberg B, Kanis JA (1997). The hospital burden of vertebral fracture in Europe: a study of national register sources. Osteoporos Int.

[38] Melton LJ, Riggs BL, Melton LJ (1995). Epidemiology of fractures. Osteoporosis: etiology, diagnosis and management, 2nd edn.

[39] Cauley JA, Chalhoub D, Kassem AM, Fuleihan G-H (2014). Geographic and ethnic disparities in osteoporotic fractures. Nat Rev Endocrinol.

[40] Karacić TP, Kopjar B (2009). Hip fracture incidence in Croatia in patients aged 65 years and more. Lijec Vjesn.

[41] Lippuner K, Johansson H, Kanis JA, Rizzoli R (2010). FRAX assessment of osteoporotic fracture probability in Switzerland. Osteoporos Int.

[42] Emaus N, Olsen LR, Ahmed LA, Balteskard L, Jacobsen BK, Magnus T, Ytterstad B (2011). Hip fractures in a city in northern Norway over 15 years: time trends, seasonal variation and mortality: the Harstad injury prevention study. Osteoporos Int.

[43] Morosano M, Masoni A, Sánchez A (2005). Incidence of hip fractures in the city of Rosario. Argentina: Osteoporos Int.

[44] Elffors L, Allander E, Kanis JA, Gullberg B, Johnell O, Dequeker J, Dilzen G, Gennari C, Lopez-Vaz AA, Lyritis G, Mazzuoli GF, Miravet L, Passeri M, Perez Cano R, Rapado A, Ribot C (1994). The variable incidence of hip fracture in southern Europe. The MEDOS study. Osteoporos Int.

[45] International Osteoporosis Foundation (2011) The Eastern European & Central Asian Regional Audit. Epidemiology, costs & burden of osteoporosis in 2010. Naturaprint, France. Available at www.iofbonehealth.org/sites/default/files/PDFs/Audit%20Eastern%20Europe_Central%20Asia/Eastern_European_Central_Asian_Audit_2010.pdf . Accessed 11 Dec 2018

[46] Kanis JA, Johansson H, Oden A, Dawson-Hughes B, Melton LJ, McCloskey EV (2010). The effects of a FRAX® revision for the USA. Osteoporos Int.

